# Risk associations of submicroscopic malaria infection in lakeshore, plateau and highland areas of Kisumu County in western Kenya

**DOI:** 10.1371/journal.pone.0268463

**Published:** 2022-05-16

**Authors:** Wilfred Ouma Otambo, Collince J. Omondi, Kevin O. Ochwedo, Patrick O. Onyango, Harrysone Atieli, Ming-Chieh Lee, Chloe Wang, Guofa Zhou, Andrew K. Githeko, John Githure, Collins Ouma, Guiyun Yan, James Kazura

**Affiliations:** 1 Department of Zoology, Maseno University, Kisumu, Kenya; 2 International Centre of Excellence for Malaria Research, Tom Mboya University College of Maseno University, Homa Bay, Kenya; 3 Department of Biology, Faculty of Science and Technology, University of Nairobi, Nairobi, Kenya; 4 Department of Population Health and Disease Prevention, University of California, Irvine, CA, United States of America; 5 Centre for Global Health Research, Kenya Medical Research Institute, Kisumu, Kenya; 6 Department of Biomedical Sciences and Technology, Maseno University, Kisumu, Kenya; 7 Centre for Global Health & Diseases, Case Western University Reserve, Cleveland, Ohio, United States of America; Ehime Daigaku, JAPAN

## Abstract

**Background:**

Persons with submicroscopic malaria infection are a major reservoir of gametocytes that sustain malaria transmission in sub-Saharan Africa. Despite recent decreases in the national malaria burden in Kenya due to vector control interventions, malaria transmission continues to be high in western regions of the country bordering Lake Victoria. The objective of this study was to advance knowledge of the topographical, demographic and behavioral risk factors associated with submicroscopic malaria infection in the Lake Victoria basin in Kisumu County.

**Methods:**

Cross-sectional community surveys for malaria infection were undertaken in three eco-epidemiologically distinct zones in Nyakach sub-County, Kisumu. Adjacent regions were topologically characterized as lakeshore, hillside and highland plateau. Surveys were conducted during the 2019 and 2020 wet and dry seasons. Finger prick blood smears and dry blood spots (DBS) on filter paper were collected from 1,777 healthy volunteers for microscopic inspection and real time-PCR (RT-PCR) diagnosis of *Plasmodium* infection. Persons who were PCR positive but blood smear negative were considered to harbor submicroscopic infections. Topographical, demographic and behavioral risk factors were correlated with community prevalence of submicroscopic infections.

**Results:**

Out of a total of 1,777 blood samples collected, 14.2% (253/1,777) were diagnosed as submicroscopic infections. Blood smear microscopy and RT-PCR, respectively, detected 3.7% (66/1,777) and 18% (319/1,777) infections. Blood smears results were exclusively positive for *P*. *falciparum*, whereas RT-PCR also detected *P*. *malariae* and *P*. *ovale* mono- and co-infections. Submicroscopic infection prevalence was associated with topographical variation (χ^2^ = 39.344, df = 2, *p*<0.0001). The highest prevalence was observed in the lakeshore zone (20.6%, n = 622) followed by the hillside (13.6%, n = 595) and highland plateau zones (7.9%, n = 560). Infection prevalence varied significantly according to season (χ^2^ = 17.374, df = 3, *p*<0.0001). The highest prevalence was observed in residents of the lakeshore zone in the 2019 dry season (29.9%, n = 167) and 2020 and 2019 rainy seasons (21.5%, n = 144 and 18.1%, n = 155, respectively). In both the rainy and dry seasons the likelihood of submicroscopic infection was higher in the lakeshore (AOR: 2.71, 95% CI = 1.85–3.95; *p*<0.0001) and hillside (AOR: 1.74, 95% CI = 1.17–2.61, *p* = 0.007) than in the highland plateau zones. Residence in the lakeshore zone (*p*<0.0001), male sex (*p* = 0.025), school age (*p* = 0.002), and living in mud houses (*p* = 0.044) increased the risk of submicroscopic malaria infection. Bed net use (*p* = 0.112) and occupation (*p* = 0.116) were not associated with submicroscopic infection prevalence.

**Conclusion:**

Topographic features of the local landscape and seasonality are major correlates of submicroscopic malaria infection in the Lake Victoria area of western Kenya. Diagnostic tests more sensitive than blood smear microscopy will allow for monitoring and targeting geographic sites where additional vector interventions are needed to reduce malaria transmission.

## Introduction

Kenya is currently ramping up malaria control interventions in order to reduce disease burden and eventually eliminate the disease. The primary vector control methods are long-lasting insecticide-treated nets (LLIN) and indoor residual spraying (IRS) [1]. Despite these efforts, existing control and treatment tools have not sufficiently reduced malaria morbidity and transmission [1, 2] and vector densities have remained high in western Kenya [3–6]. A substantial proportion of *Plasmodium* infections are submicroscopic, with densities too low to be detected using standard diagnostic methods such as blood smear microscopy. Concerns have been raised that asymptomatic submicroscopic infections sustain ongoing *Plasmodium* transmission, particularly if microscopy is used exclusively for *Plasmodium* parasite detection [7, 8].

Topography is a major determinant of malaria transmission and endemicity because it affects local hydrology and the stability and productivity of vector breeding habitats. It is also linked to the spatial distribution of malaria exposure in the human population [9]. Local populations may be vulnerable to weather-driven epidemics in areas with low transmission [9–13]. The topography of highland regions in western Kenya consist of hills, valleys, and plateaus with various drainage characteristics [10]. Mosquito breeding habitats are widely distributed in lowland areas, whereas breeding habitats are sparse in highlands due to efficient drainage. The heterogeneous distribution of larval breeding sites also likely affects adult vector spatial distribution and the risk of human exposure to infective mosquitoes [9, 10, 14]. Despite the effect of topography on *Plasmodium* infection risk, information regarding variables that may contribute to submicroscopic infection throughout the year in western Kenya near the Lake Victoria basin is meager.

Seasonality has also been shown to influence malaria burden, with rainfall having a positive correlation with infection prevalence in both wet and dry seasons. Declines in transmission observed in prolonged dry seasons are most likely due to the reduced numbers of vector breeding sites [15]. The highland plateau region of western Kenya near the Lake Victoria basin is located at an elevation of 1600m above sea level, whereas the elevation near the shore is significantly lower. Numerous valleys and basin-like depressions with varying levels of malaria transmission intensity occur in hillside regions located between the basin of Lake Victoria and highland plateau. Human settlements along the hillside depressions may serve as the main malaria reservoir during the dry seasons [3, 11, 15–17]. However, the overall impact of seasonality on submicroscopic infection prevalence in these areas remains undetermined. The objective on this study was to evaluate topological, demographic and behavioral risk factors that correlate with community submicroscopic malaria infection prevalence in these regions of Kisumu County, Kenya.

## Materials and methods

### Study area and population

The study was carried out in the Nyakach Sub-County of Kisumu County in western Kenya near the shore of Lake Victoria at latitude -.333333° and longitude 34.991°. The area includes three eco-epidemiological zones: lakeshore, hillside and highland plateau. The lakeshore zone is characterized by a flat plain at an elevation of 1100-1200m above sea level. It is prone to flooding during the rainy season. The highland plateau has elevations ranging from 1450 to >1600m. The hillside zone is located at 1300-1450m, between the lakeshore and Nyabondo highland plateau. Larval habitats are unstable and permanent habitats scarce in this zone. Nyakach sub-County has an area of approximately 327 square kilometers and a population of 168,140 people living in 35,553 households at a population density of 460 people per square km [18].

### Study participation and data collection

Cross-sectional community based surveys were performed in June and November in 2019 and 2020. Malaria transmission generally peaks after the long rainy season in June. The dry season in November is associated with minimal transmission in western Kenya [14, 19]. A demographic surveillance system was used to map house locations and conduct a population census at the beginning of the study. The survey was conducted during the Covid-19 era, however, all infection prevention and control protocols were followed according to Ministry of Health guidelines [20]. Field assistants and community health volunteers (CHVs) were recruited from the local community and trained on the use of Open Data Kit (ODK) software in an Android Samsung tablet (Version SAM-T380) with a questionnaire for entering data requirements following an interview with the household head. The survey sought to determine community health, demographic, and socioeconomic characteristics. The questionnaire collected self-reported information on age, gender, LLIN use, household structure, primary occupation, and fever history. Participants were categorized into three age groups (<5 years old, 5–<15 years old, and ≥15 years old). LLIN use was defined as sleeping under a bed net the night before the survey. The wall material type was used to assess house structure categorized into the following groups: Brick/Block, Mud & Wood, and Mud & Cement. Occupation was divided into four categories (farmer, commercial sales, child younger than working age, and unemployed). Following the WHO definition, a febrile case was defined as an individual with an axillary temperature ≥37.5°C at the time of examination or complaints of fever and other non-specific constitutional symptoms prior to examination [21].

A total of 1,777 finger-prick blood smears and filter paper DBS samples were collected for parasite examination via microscopy and RT-PCR, respectively. Blood samples were transported to the International Centre of Excellence for Malaria Research (ICEMR) laboratories at Tom Mboya University College located in Homa Bay, Kenya for further analysis.

### Processing of blood smears

Finger prick blood smears were examined for *Plasmodium* parasites by microscopic inspection. The thick and thin blood films were stained with 10% Giemsa. Two expert microscopists examined the slides in immersed oil to identify the parasite species. If at least one asexual blood-stage malaria parasite was found on a slide, it was considered positive. Discrepancies in slide readings were confirmed by a third experienced microscopist for quality control.

### DNA extraction and screening for *Plasmodium* parasite

The Chelex resin (Chelex-100) saponin method was used with minor modifications [22]. Primers and probes specific to *Plasmodium* species were used to target 18S ribosomal RNA [23]. Six μL of PerfeCTa^®^ qPCR ToughMix^™^, Low ROX^™^ Master mix (2X), 0.4 μL each of the forward and reverse species-specific primers (10 μM), 0.5 μL of the species-specific probe, 0.1 μL of double-distilled water and 2 μL of parasite DNA were used in the PCR reaction. The following RT-PCR cycling conditions were used: 50°C for 2 minutes, (95°C for 2 minutes, 95°C for 3 seconds, and 58°C for 30 seconds) for 45 cycles (QuantStudio^™^ 3 Real-Time PCR System). Nested PCR to detect *P*. *vivax* DNA was not performed since recent studies of finger prick blood samples from ~1,000 local residents failed to detect a single *P*. *vivax* infection. Submicroscopic malaria infection was defined as an infection detected by RT-PCR but not by microscopy.

### Ethical considerations

The study received ethical approval from Maseno University’s Ethics Review Committee (reference number: MSU/DRPI/MUERC/00778/19) and the University of California, Irvine’s Institutional Review Board (HS# 2017–3512). Administrative approval to conduct the study was obtained from the Kisumu County Director of Health and the Nyakach Deputy County Commissioner. The survey was open to all community residents who were willing to participate in the study. Residents who declined to participate in the study or changed their willingness to participate at any time were excluded from the study analysis. All study participants provided written informed consent. Minors provided assent with informed consent from parents or guardians.

### Data analysis

IBM SPSS Software Version 21.0 was used to analyze the data. The Chi-square test was used to assess the significance association in malaria infection prevalence across seasons and topography. Multiple comparisons between *Plasmodium* species and seasonality were performed using the Kruskal–Wallis test followed by Dunn’s multiple comparison test. The adjusted agreement between microscopy and RT-PCR results were measured using Cohen’s kappa statistic, sensitivity, specificity, positive predicted value, negative predicted value, and diagnostic accuracy. Univariate binary logistic regression and multivariate mixed effect binary logistic regression analyses were used to identify the risk factors associated with submicroscopic infection prevalence. In the univariate analysis, all risk factors were tested, and only those with *p*≤0.50 were chosen and included in the multivariate analysis. In the multivariate analysis, variables with *p*<0.05 were considered significant risk factors. Topography, gender, age, bed net usage, wall type, occupational, education level, household population size, bednet type, symptoms, and seasonality were among the variables investigated. Chi-square test and odds ratio (OR) with p values <0.05 were considered significant.

## Results

Cross-sectional community based surveys were conducted in the June 2019 and June 2020 wet seasons (n = 458 and n = 388 study participants, respectively) and the November 2019 and November 2020 dry seasons (n = 456 and n = 475, respectively).

### Demographic characteristics of the study population

There were no differences in the number of study participants residing in the lakeshore, hillside and highland plateau topographical areas (*p =* 0.667) (Table 1). Study participants were more frequently female than male (*p* = 0.039), equal to and older than 15 years (*p*<0.0001), and occupied a household that included three or less residents (*p*<0.0001). Reported use of long lasting insecticide treated bed nets was 95.4% at the time of study enrollment. There were minor changes in bed net use according to season and year, but self-reported bed net use was greater than 90% in all four cross-sectional surveys (Table 1).

**Table 1:** Geographic and demographic characteristics of study population.

Parameter		Enrollment		Season								*p*-value
				Rainy (June-19)		Dry (Nov-19)		Rainy (June-20)		Dry (Nov-20)		
				N = 458		N = 456		N = 388		N = 475		
		n	%	n	%	n	%	n	%	n	%	
**Topography of residence area**	Lakeshore	622	35.0	155	33.8	167	36.6	144	37.1	156	32.8	0.667
	Hillside	595	33.5	151	33.0	158	34.6	123	31.7	163	34.3	
	Plateau	560	31.5	152	33.2	131	28.7	121	31.2	156	32.8	
**Sex**	Male	674	37.9	160	65.1	181	39.7	167	43	166	34.9	0.039
	Female	1103	62.1	298	34.9	275	60.3	221	57	309	65.1	
**Age**	<5 years	241	13.6	75	16.4	50	11.0	36	9.3	80	16.8	<0.0001
	5—<15 years	533	30.0	99	21.6	158	34.6	135	34.8	141	29.7	
	≥15 years	1003	56.4	284	62.0	248	54.4	217	55.9	254	53.5	
**Household size**	≤ 3 individuals	849	47.8	267	58.3	154	33.8	164	42.3	264	55.6	<0.0001
	4–5 individuals	691	38.9	163	35.6	180	39.5	175	45.1	173	36.4	
	>5 Individuals	237	13.3	28	6.1	122	26.8	49	12.6	38	8.0	
**Education level**	Never attended school	88	5.0	10	2.2	31	6.8	28	7.2	19	4.0	<0.0001
	Younger than school age	164	9.2	54	11.8	40	8.8	23	5.9	47	9.9	
	Primary school	952	53.6	187	40.8	283	62.1	223	57.5	259	54.5	
	Secondary school	458	25.8	173	37.8	78	17.1	93	24	114	24.0	
	College & above	115	6.5	34	7.4	24	5.3	21	5.4	36	7.6	
**Occupation/ income generating activity**	Farmer	521	29.3	177	38.6	135	29.6	70	18.1	139	29.3	<0.0001
	Commercial sales	252	14.2	70	15.3	54	11.8	74	19.1	54	11.4	
	Unemployed	104	5.9	36	7.9	24	5.3	21	5.4	23	4.8	
	Child younger than working age	900	50.6	175	38.2	243	53.3	223	57.4	259	54.5	
**Bed net use**		1696	95.4	418	91.3	445	97.6	367	94.6	466	98.1	<0.0001

### Submicroscopic malaria infection prevalence and correlation with topographical zone and seasons

Out of the 1,777 samples, submicroscopic infection was 14.2% (253/1,777), with microscopy and RT-PCR detecting 3.7% (66/1,777) and 18% (319/1,777) infections, respectively (Table 2). The overall prevalence of *Plasmodium* parasitemia detected by microscopic inspection of blood smears and RT-PCR, respectively, varied from 2.3% to 4.8% (*p* = 0.2355) and 12.8% to 24.6% (*p*<0.0001) over the two rainy and dry seasons. Blood smear microscopy exclusively identified *P*. *falciparum* infections, whereas RT-PCR identified *P*. *malariae* and *P*. *ovale* mono-infections and co-infections with *P*. *falciparum*. *Plasmodium malariae* and *P*. *ovale* were detected in less than one percent of blood samples (Table 2). Submicroscopic infection were significantly different across seasonality (χ^2^ = 17.374, df = 3, *p*< 0.0001) with high infection in the dry season of November 2019 (19.7%, n = 456), followed by the rainy season of June 2020 (13.9%, n = 458), then the rainy season of June 2019 (12.9, n = 458), and the dry season of November 2020 (10.5%, n = 475) (Table 2).

**Table 2:** Plasmodium infection prevalence according to season and diagnostic test.

Diagnosis	Plasmodium species	Season				Chi-square value^1^	*p*-value
		Rainy (June-19) Infection (95% CI)	Dry (Nov-19) infection (95% CI)	Rainy (June-20) infection (95% CI)	Dry (Nov-20) infection (95% CI)		
Number of study participants		458	456	388	475		
Microscopy Percent positive (95% CI)	***P*. *falciparum***	3.9 (2.1, 5.7)	4.8 (2.9, 6.8)	3.8 (1.9, 5.7)	2.3 (1.0, 3.7)	4.252	0.2355
	***P*. *malariae***	0	0	0	0		NA^2^
	***P*. *ovale***	0	0	0	0		NA
	***P*. *falciparum +*** ***P*. *malariae***	0	0	0	0		NA
	***P*. *falciparum +*** ***P*. *ovale***	0	0	0	0		NA
	**Total**	3.9 (2.1, 5.7)	4.8 (2.9, 6.8)	3.8 (1.9, 5.7)	2.3 (1.0, 3.7)	4.252	0.2355
RT-PCR Percent positive (95% CI)	***P*. *falciparum***	15.3 (12.0, 18.6)	23.9 (20.0, 27.8)	15.7 (12.1, 19.4)	11.4 (8.5, 14.2)	27.80	<0.0001
	***P*. *malariae***	0.4 (0.17, 1.04)	0.2 (0.21, 0.65)	0.4 (0.17, 1.04)	0.2 (0.20, 0.62)	0.706	0.8717
	***P*. *ovale***	0.2 (0.21, 0.64)	0.4 (0.16, 1.00)	0.6 (0.09, 1.40)	0.6 (0.08, 1.35)	1.178	0.7584
	***P*. *falciparum +*** ***P*. *malariae***	0.4 (0.16, 1.04)	0	0.2 (0.21, 0.65)	0.4 (0.16, 1.01)	2.156	0.5407
	***P*. *falciparum +*** ***P*. *ovale***	0.4 (0.16, 1.04)	0	0.5 (0.20, 1.23)	0.2 (0.20, 0.62)	2.522	0.4714
	**Total**	16.8(12.2, 19.8)	24.6 (20.4, 28.2)	17.8(15.6, 22.9)	12.8(9.8, 16.0)	21.68	<0.0001
Percent submicroscopic infections (95% CI)		12.9 (9.8, 16.0)	19.7 (16.0, 23.4)	13.9 (10.5, 17.4)	10.5 (7.8, 13.3)	17.36	0.0006

^1^ Kruskal-Wallis H test

^2^ NA = Not Applicable

**Fig 1:**
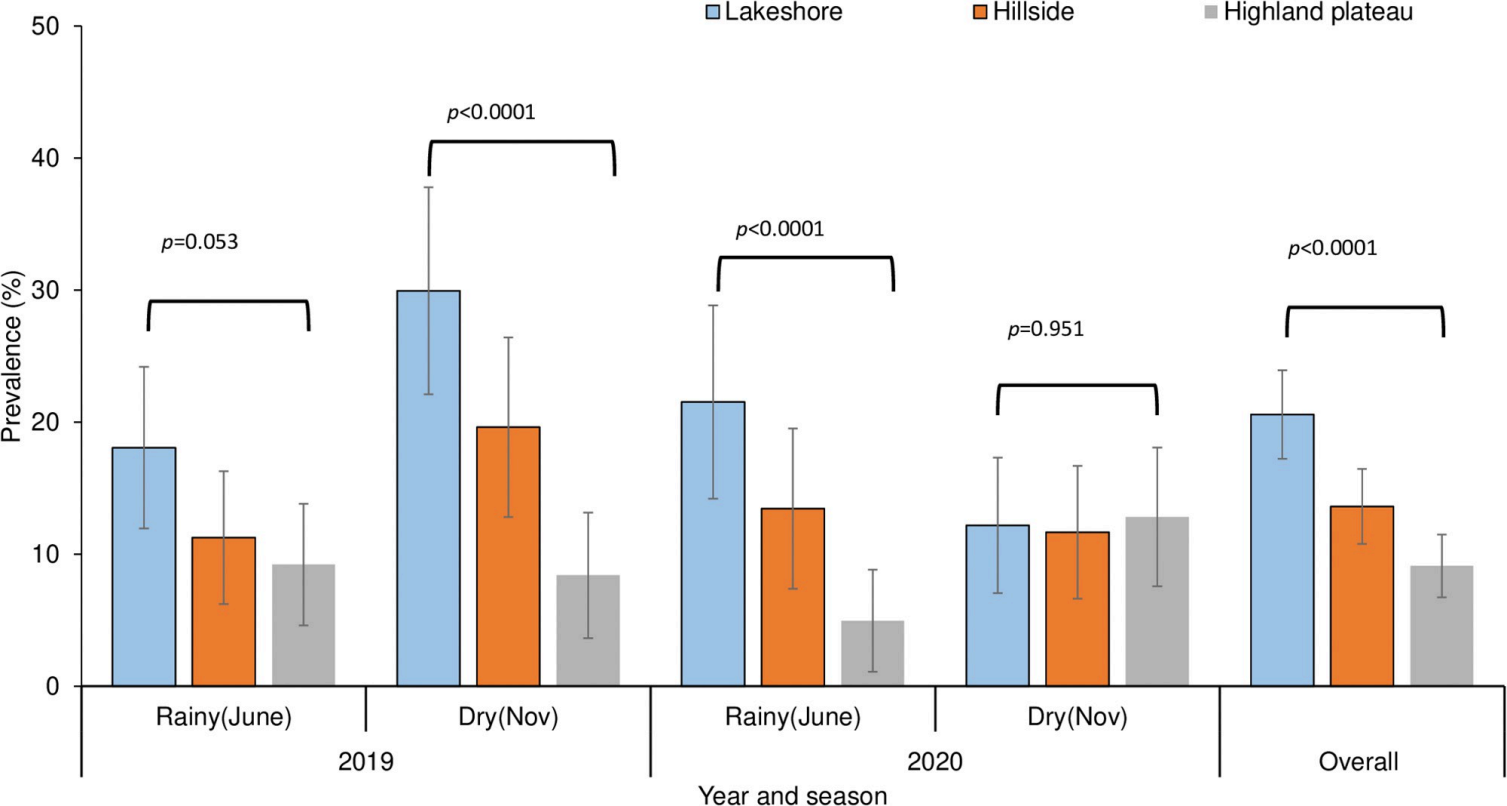
Submicroscopic malaria infection prevalence across topographic zones in rainy and dry seasons.

Submicroscopic *P*. *falciparum* infection prevalence showed significant topographical variation (χ^2^ = 39.344, df = 2, *p*<0.0001), with highest value in the lakeshore zone (20.6%) followed by the hillside zone (13.6%) and highland plateau zone (9.1%) (Fig 1). Further post-hoc multiple comparison analysis revealed significant variation within each and every topographical site (p<0.05) (S1 Table). The highest sub-microscopic prevalence was observed in residents of the lakeshore zone in the 2019 dry season (29.9%, n = 167) and 2020 and 2019 rainy seasons (21.5%, n = 144 and 18.1%, n = 155, respectively) as compared to the hillside and the highland plateau zones (Fig 1).

### Risk factors associated with submicroscopic malaria infection

Residency in the lakeshore zone, age ≤4 years and 5-<15 years, male sex, household wall type consisting of mud and cement, subjective fever and elevated body temperature recorded at the time finger prick blood sample was obtained, and the 2019 dry season were associated with an increased risk of submicroscopic malaria infection by univariate analysis (Table 3). All risk factors were tested in the univariate analysis, and only those with P≤0.50, such as topography, gender, age, bed net usage, wall type, occupational, education level, household population size, bednet type, symptoms, and seasonality, were selected and included in the multivariate analysis. The variables topography, gender, age, bed net usage, wall type, occupational, symptoms, and seasonality qualified for inclusion in the multivariate analysis. Multivariate analysis revealed that the associations were significant. However, bed net use was not correlated with submicroscopic infection prevalence (Table 3).

**Table 3:** Predictive factors associated with submicroscopic malaria infection.

Risk factor	Category	Submicroscopic infection n (%)	Univariate		Multivariate	
			OR (95% CI)	*p*-value^1^	AOR (95% CI)	*p*-value^2^
Topography of residence area	Lakeshore	128 (20.6)	3.04 (2.11, 4.37)	<0.0001	2.71 (1.85, 3.95)	<0.0001
	Hillside	81 (13.6)	1.85 (1.26, 2.72)	0.002	1.74 (1.17, 2.61)	0.007
	Plateau	44 (7.9)	1			
Age group	<5 years	19 (7.9)	0.58 (0.35, 0.96)	0.034	0.54 (0.322, 0.89)	0.017
	5-<15 years	105 (19.7)	1.66 (1.25, 2.20)	<0.0001	1.57 (1.17, 2.09)	0.002
	≥15 years	129 (12.9)	1			
Sex	Female	141 (12.8)	0.74 (0.56, 0.96)	0.025	0.74 (0.56, 0.96)	0.025
	Male	112 (16.6)	1			
Bed net usage	No net	14 (17.9)	1.34 (0.74, 2.42)	0.339	1.66 (0.88, 3.10)	0.115
	Use net	239 (14.1)	1			
Wall type	Brick & block	40 (11.4)	0.61 (0.39, 0.95)	0.030	0.64 (0.41, 1.00)	0.049
	Mud & wood	160 (14.2)	0.78 (0.55, 1.09)	0.144	0.63 (0.42, 0.98)	0.044
	Mud & cement	53 (17.6)	1			
Occupation/ income generating activity	Farmer	60 (11.5)	0.70 (0.46, 1.06)	0.098	0.60 (0.39, 0.93)	0.023
	Commercial sales	27 (11.5)	0.70 (0.42, 1.16)	0.168	0.61 (0.36, 1.04)	0.070
	Child younger than working age	47 (14.9)	1.06 (0.73, 1.54)	0.772	0.77 (0.46, 1.29)	0.314
	Unemployed	119 (16.9)	1			
Symptoms	Asymptomatic	189 (12.7)	0.51 (0.37, 0.70)	<0.0001	0.69 (0.48, 0.99)	0.048
	Fever	64 (22.1)	1			
Seasonality	Wet (June 2019)	59 (12.9)	1.26 (0.84, 1.88)	0.263	1.20 (0.78, 1.83)	0.416
	Dry (November 2019)	90 (19.7)	2.09 (1.44, 3.03)	<0.0001	1.69 (1.12, 2.54)	0.012
	Wet (June 2020)	54 (13.9)	1.37 (0.91, 2.07)	0.129	1.41 (0.85, 2.35)	0.182
	Dry (November 2020)	50 (10.5)	1			

^1^ P-value determined using univariate binary logistic regression model.

^2^ P-value determined using multivariate mixed effects binary logistic regression model-variables with P value <0.50 in the unadjusted unvariate analysis were considered.

### Impact of topography on risk factors associated with submicroscopic malaria infection

Children under the age of 5 years old in the lakeshore zone had a lower risk of submicroscopic infection than residents over the age of 15 years old (AOR: 0.23, 95% CI = 0.10–0.57, *p* = 0.002). Furthermore, school-aged children (5-<15 years old) in the hillside (RR: 0.56, 95%CI = 0.39–0.82, *p* = 0.002) and highland plateau zones (RR: 0.25, 95% CI = 0.14–0.45, *p*<0.0001) had a lower risk of submicroscopic infection than those in the lakeshore zone (Table 4). In the lakeshore zone, females had a lower likelihood of submicroscopic infection than males (AOR: 0.67, 95% CI = 0.45–0.98, *p* = 0.042), but the hillside (*p* = 0.102) and highland plateau (*p* = 0.812) zones had no effect on gender likelihood of submicroscopic infection (Table 4). The relative risk of submicroscopic infection among females was lower in the hillside (RR: 0.66, 95% CI 0.46–0.93, *p* = 0.018) and highland plateau (RR: 0.45, 95% CI = 0.30–0.68, *p*<0.0001) zones compared to the lakeshore zone. Males in the hillside (RR: 0.67, 95% CI = 0.46–0.97, *p* = 0.030) and highland plateau (RR: 0.30, 95% CI = 0.30–0.68, *p*<0.0001) zones were less likely to be infected than males in the lakeshore zones (Table 4). Although people who did not use bed nets in the highland plateau zone had higher likelihood of submicroscopic infections than those who did (AOR: 3.37, 95% CI = 1.19–9.54, *p* = 0.022), there was no significant difference in the likelihood of infection between bednet users and non-users in the lakeshores (*p* = 0.523) and hill side zones (*p* = 0.958) (Table 4). The risk of sub-microscopic infection among bednet users was lower in the hillside (RR: 0.67, 95% CI = 0.52–0.87, *p* = 0.002) and highland plateau zones (RR: 0.35, 95% CI = 0.25–0.50, *p*<0.0001), than in the lake zone. Residency in mud wall houses on the hillside and highland plateau had a lower risk of submicroscopic infection than those who lived in lakeshore zones, with risk factors of 0.48 (RR: 0.48, 95% CI = 0.34–0.67, *p*<0.0001) and 0.38 (RR: 0.38, 95% CI = 0.26–0.57, *p*<0.0001), respectively (Table 4). The likelihood of the submicroscopic infection being symptomatic or asymptomatic were the same in the lakeshore (*p* = 0.0.613), hillside (*p* = 0.125) and the highland plateau (*p* = 0.710) (Table 4). The rainy and dry seasons had a significant impact on the infection across the topographic zones. In the lakeshore zone, the rainy season of 2020 had 3.4 times the odds of submicroscopic infection than the dry season (AOR = 3.40, 95% CI = 1.57–7.36, *p* = 0.002).

**Table 4:** Influence of topography on risk factors associated with submicroscopic malaria infection.

Risk factor	Category	Lakeshore zone^1^			Hillside zone			Highland plateau zone			Risk ratio^2^			
		Infection (n, %)	AOR (95% CI)^1^	*p*-value	Infection (n, %)	AOR (95% CI) ^1^	*p*-value	Infection (n, %)	AOR (95% CI) ^1^	*p*-value	Hillside (95% CI)^2^	*p*-value	Plateau (95% CI)^2^	*p*-value
Age	0–4 years	9 (10.6)	0.23 (0.10, 0.57)	0.002	6 (7.7)	0.49 (0.18, 1.28)	0.149	4 (5.1)	0.57 (0.16, 2.01)	0.381	0.73 (0.27, 1.95)	0.522	0.48 (0.16, 1.51)	0.199
	5-<15 years	61 (31.0)	1.02 (0.57, 1.83)	0.943	32 (17.5)	1.14 (0.62, 2.13)	0.673	12 (7.8)	0.96 (0.41, 2.24)	0.915	0.56 (0.39, 0.82)	0.002	0.25 (0.14, 0.45)	<0.0001
	≥15 years	58 (17.1)	1		43 (12.9)	1		28 (8.5)	1		0.75 (0.52, 1.09)	0.128	0.50 (0.33, 0.76)	0.001
Sex	Female	69 (18.0)	0.67 (0.45, 0.98)	0.042	44 (11.8)	0.67 (0.42, 1.08)	0.102	28 (8.1)	1.08 (0.57, 2.05)	0.812	0.66 (0.46, 0.93)	0.018	0.45 (0.30, 0.68))	<0.0001
	Male	59 (24.8)	1		37 (16.6)	1		16 (7.5)	1		0.67 (0.46, 0.97)	0.030	0.30 (0.18, 0.51)	<0.0001
Bed net usage	No net	6 (24.0)	1.39 (0.50, 3.8)	0.523	2 (11.1)	1.04 (0.22, 5.00)	0.958	6 (17.1)	3.37 (1.19, 9.54)	0.022	0.46 (0.11, 2.03)	0.502	0.71 (0.26, 1.96)	0.512
	Use net	122 (20.4)	1		79 (13.9)	1		38 (7.2)	1		0.67 (0.52, 0.87)	0.002	0.35 (0.25, 0.50)	<0.0001
Wall type	Block	17 (15.9)	0.91 (0.43, 1.91)	0.798	13 (17.1)	1.18 (0.53, 2.65)	0.682	10 (6.0)	0.55 (0.15, 2.06)	0.375	1.12 (0.61, 2.25)	0.823	0.38 (0.18, 0.79)	0.007
	Mud & wood	90 (22.3)	1.42 (0.81, 2.50)	0.221	40 (10.7)	0.68 (0.37, 1.24)	0.209	28 (8.6)	0.73 (0.23, 2.37)	0.601	0.48 (0.34, 0.67)	<0.0001	0.38 (0.26, 0.57)	<0.0001
	Mud & Cement	21 (18.8)	1		28 (19.2)	1		6 (9.3)	1		1.02 (0.61, 1.70)	0.920	0.50 (0.18, 1.36)	0.152
Symptoms	Asymptomatic	86 (18.4)	0.85 (0.49, 1.46)	0.558	63 (12.2)	0.56 (0.27, 1.16)	0.118	34 (7.1)	1.18 (0.37, 3.78)	0.776	0.66 (0.49, 0.89)	0.125	0.54 (0.40, 0.75)	<0.0001
	Symptomatic	42 (27.1)	1		18 (23.1)	1		10 (7.1)	1		0.29 (0.19, 0.44)	<0.0001	0.09 (0.03, 0.23)	<0.0001
Occupation/ income generating activity	Farmer	28 (15.9)	0.37 (0.18, 0.75)	0.006	16 (9.2)	0.54 (0.25, 1.15)	0.109	16 (9.4)	1.36 (0.55, 3.35)	0.500	0.58 (0.32, 1.03)	0.058	0.59 (0.33, 1.05)	0.067
	Commercial sales	9 (13.0)	0.27 (0.11, 0.68)	0.005	15 (16.1)	1.11 (0.48, 2.56)	0.805	6 (4.1)	0.58 (0.15, 2.06)	0.171	0.59 (0.33, 1.05)	0.584	0.32 (0.09, 1.12)	0.056
	Child younger than working age	71 (25.0)	0.31 (0.14, 0.68)	0.003	35 (15.1)	1.04 (0.42, 2.57)	0.927	16 (7.1)	3.71 (1.00, 13.79)	0.050	0.60 (0.42, 0.86)	0.006	0.28 (0.17, 0.48)	<0.0001
	Unemployed	20 (21.5)	1		15 (15.6)	1		9 (9.9)	1		0.73 (0.40, 1.33)	0.299	0.46 (0.22, 0.96)	0.030
Season	Wet (2019)	28 (18.1)	1.5 (0.77, 3.05)	0.225	17 (11.3)	1.11 (0.50, 2.46)	0.795	14 (9.2)	0.68 (0.28, 1.70)	0.414	0.78 (0.49, 1.25)	0.296	0.30 (0.14, 0.60)	0.0002
	Dry (2019)	50 (29.9)	2.54 (1.26, 5.09)	0.009	31 (19.6)	1.69 (0.84, 3.42)	0.143	9 (6.9)	0.77 (0.32, 1.83)	0.548	0.57 (0.35, 0.92)	0.018	0.42 (0.23, 0.74)	0.002
	Wet (2020)	31 (21.5)	3.4 0 (1.57, 7.36)	0.002	17 (13.8)	1.11 (0.43, 2.90)	0.829	6 (5.0)	0.16 (0.04, 0.63)	0.009	0.56 (0.31, 1.00)	0.049	0.25 (0.11, 0.57)	0.0003
	Dry (2020)	19 (12.2)	1		16 (9.8)	1		15 (9.6)	1		0.74 (0.43, 1.25)	0.251	1.19 (0.61, 2.32)	0.610

^1^ Multivariate binary logistic regression model used for risk factor analysis.

^2^ Significance by χ2 test for risk ratio of submicroscopic malaria infection according to topographic zone with Lakeshore zone as referent.

In the highland plateau zone, the wet season of 2020 had 0.16 times odds of submicroscopic infection as the dry season of 2020 (AOR: 0.16, 95% CI = 0.04–0.63, *p* = 0.009) (Table 4).

### Risk factors associated with submicroscopic infection in malaria infection

A follow-up analysis was performed to determine the prevalence of submicroscopic infection among the RT-PCR positive cases. Risk factors for submicroscopic infection were also identified among the RT-PCR positives, including topography, age, gender, wall type, occupation, symptoms, and seasonality. The sub-microscopic infection was found in 79.3% (253/319) of the malaria infections. Among the RT-PCR positives, only age group was significantly associated with submicroscopic infection (*p*<0.0001). Topography, gender, wall type, occupation, symptoms, and seasonality, on the other hand, were not associated with submicroscopic infection in the malaria infection (S2 Table).

## Discussion

Despite increased vector control efforts, the persistence of infection in endemic areas remains problematic. The submicroscopic infection as well as asymptomatic infection in the population serve as a reservoir for infectious gametocytes, resulting in continuous malaria transmission. The current study provides evidence on submicroscopic infection carriage along a transect in western Kenya from the lowland Lake Victoria basin through the hillside to the highland plateau zones, as well as the influence of seasonality and topography on risk factors associated with submicroscopic infection. The submicroscopic infection in the study area was high at 14.2% (N = 1,777) with the overall prevalence by microscopy and RT-PCR at 3.7% (N = 1,777) and 18% (N = 1,777) despite the bed net coverage of 95%. The infection showed topographical and seasonal variations with lower altitudes areas and the wet seasons having the highest carriage of infection. In addition, there was a strong association with carriage of submicroscopic infection if one lived in the lowland lake zone, or was a male, or was of school going age and lived in a mud walled house. Bed net ownership and resident’s economic activity did not influence the carriage of submicroscopic infection.

The prevalence of submicroscopic infection varied along the altitudinal transect, according to the current study. When compared to the highland plateau zone, the lakeshore zone, which is adjacent to Lake Victoria, had 3-fold higher likelihood of submicroscopic infection. The findings are consistent with other studies that show a higher prevalence of malaria in lowland areas than in highland areas [9–13]. The lowland lakeshore zone is distinguished by a flat plain frequented by flooding, resulting in stagnant water bodies that are potential mosquito breeding habitats, whereas transmission in the highland plateau area may be less distinct due to the flat topography and the more diffuse hydrology resulting from numerous streams, hence better drainage and lower stability of malaria transmission [9, 24–26]. Similarly, residents of the hillsides zones were found to be twice as likely as those of the highland plateau zone to have submicroscopic infections (AOR: 1.74, 95% CI = 1.17–2.61, *p* = 0.007). As rivers and streams run along the valley bottoms and the majority of breeding habitats are confined to the valley bottoms as hillside gradients provide efficient drainage, the hillside zone has unstable larval habits. As a result, heterogeneous distribution of larval breeding habitats is likely to affect adult vector distribution and heterogeneity exposure of the human population to malaria [9, 14]. In addition, rainfall correlates positively with malaria incidence, and malaria distribution varies across different levels of geographical elevation [27]. The hillside zone was discovered to have man-made ponds and constructed water pans that take time to dry out, providing potential breeding sites for mosquitos throughout the seasons. Despite the fact that the hillside is always dry, human activity on the land, such as the construction of ponds for water reservoirs, serves as a potential breeding site for mosquitoes. Pools of water collect in the lowlands zones, and flooding occurs frequently, resulting in stagnant water bodies that are potential mosquito breeding sites, as opposed to the hillside and highland plateau, which have efficient natural drainage and diffuse hydrology as a result of numerous streams [9, 10, 14, 28]. During the rainy season, vectors migrate from valley bottoms towards the top of the hills, being the reason for the increasing malaria prevalence at higher altitudes [12]. Studies have reported high habitat productivity during the rainy season correlates with increased vector density and species richness [14, 29, 30], however, the current study did not investigate the effect of seasonality on larval habitats productivity and the infection burden.

Malaria is expected to be present throughout the year in endemic areas of western Kenya, with seasonal peaks during the rainy season [15, 19, 31–34]. Environmental modification caused by economic activities such as dam constructions, where large reservoirs appear to facilitate malaria transmission by increasing breeding habitat for malaria vector mosquitoes, appear to facilitate malaria transmission [35]. Environmental manipulation was observed in the study areas as a result of economic activities such as brick-making, fish farming, irrigation, rock mining, water pans and man-made ponds that leave depressions on the grounds that are filled with water could explain the persistence of the infections in the study area. Pools of stagnant water are vector breeding grounds, so even if it doesn’t rain, conditions that mimic rainy seasons are available even during the dry season, allowing pools of water to stay wet for longer periods of time without drying up, resulting in more vector breeding sites. Climate variability, which has been shown to influence malaria transmission in endemic areas, may also play a role in infection persistence because the infectious vector population may increase under favorable climatic conditions, resulting in malaria prevalence rates remaining stable [3, 15, 33, 36–39].

Existing control and treatment tools have not suppressed mosquito-borne *P*. *falciparum* infection and disease transmission, necessitating the scaling up of intervention strategies [1]. Despite the high net coverage of 95%, not using a bed net had no statistically significant effect on the likelihood of having submicroscopic infections (*p* = 0.115). The findings contradict previous research that found long-lasting insecticide-treated nets to be especially important in reducing malaria incidence [40–42]. Furthermore, while people who did not use bed nets in the highland plateau zone had an increased risk of submicroscopic infections compared to bed net users (AOR: 3.37, 95% CI = 1.19–9.54, *p* = 0.022), those who did not use bed nets in the lake and hillside zones had equal chances of infection. Non-compliance with LLINs is linked to an increased risk of malaria [42]. However, the current study found no difference in submicroscopic infection between bednet users and non-bednet users (*p* = 0.115), which could be due to behavioral changes in malaria vectors, with outdoor transmission occurring earlier in the evenings and mornings when people are not protected by LLIN [43–45], and increased insecticide resistance to insecticides used in LLINs [46], though the current study did not investigate these factors.

In the current study, school-aged children were 1.5 times more likely than ≥15 year olds to have sub-patent infection. The current study supported previous research that found that school-going children had an increased risk of submicroscopic infection, acting as reservoirs of infectious gametocytes and thus maintaining infection in the community [7, 47]. This higher prevalence of submicroscopic infections in school-age children could be attributed to increased *Plasmodium* exposure as a result of inadequate bed net use [8]. Individuals in malaria-endemic areas develop adaptive immunity as a result of frequent exposure to the *P*. *falciparum* parasite, and this is related to age [48]. Adults are asymptomatic parasite carriers because they have developed strong immunity to malaria parasites through repeated exposures, whereas young children are frequently symptomatic because their anti-malarial immunity is still developing [7]. Furthermore, school-aged children from the hillside and highland plateau zones had a lower risk of submicroscopic infection than those from the lakeshore zones. The lower transmission pressure on the highland plateau may make residents more susceptible to *Plasmodium* infection, as children on the highland plateau may have a slower ability to suppress parasite density than children in lowland areas with well-established transmission [9, 47]. While people living in and near lowland areas have a large reservoir of infectious gametocytes, people living away from this area have a high proportion of susceptible individuals to patent malaria.

Gender bias in infections could be attributed to socioeconomic differences that kept them awake late at night and early in the morning, adult males engage in nocturnal outdoor gatherings without the protection of the IRS and treated nets, exposing themselves to mosquito bites [8, 49]. This could explain why females were 0.7 times less likely than males to develop submicroscopic infection in the current study (AOR: 0.74, 95% CI = 0.56–0.96, *p* = 0.025). According to the current study, doing business during the dry season increased the risk of developing submicroscopic infection by 2.68 times. This could be because of increased exposure to infection risk as a result of favorable weather, as opposed to business disruption caused by rain, which caused the business to close early. The main economic activity in Lakeshore zone is fishing and reed cutting for mats, which causes residents to stay close to water for longer periods of time, exposing themselves to mosquito breeding sites, resulting in higher biting rates and high infection levels. The main economic activity in the highland plateau is farming and brick-making. The brick-making sites have pits that, when filled with water, provide potential breeding grounds for mosquitoes. However, further investigation revealed that the type of occupation had no effect on the likelihood of developing submicroscopic infection across the topography.

The current study found that socio-demographic factors such as housing type was linked to submicroscopic infection. The characteristics of the house have an impact on submicroscopic infection. The current study discovered that houses constructed with mud, or mud and cement increased the risk of submicroscopic infection, which is consistent with the findings of other studies [19, 25, 26]. The majority of residents in the lakeshore zone had poor housing quality, with traditionally constructed housing being common. Low socioeconomic status, and poor housing quality may correlate with the use of personal protection measures, resulting in an increased risk of infection [50]. The rapid emergence and spread of the COVID-19 has resulted in massive global disruptions that are affecting people’s lives and well-being. The devastation caused by the pandemic could be greatly exacerbated if the response jeopardizes the provision of life-saving malaria services [51]. COVID-19-related challenges have contributed to an increase in antimalarial and RDT stockout rates, resulting in a drop in test-and-treat policy adherence. [52]. Reduced funding for vector interventions, combined with competing public health challenges such as the ongoing COVID-19 pandemic, may result in a rollback of malaria control gains, leading to increased morbidity and mortality from malaria [53–55].

The current study blood smear microscopy exclusively identified *P*. *falciparum* infections, whereas RT-PCR identified *P*. *malariae* and *P*. *ovale* mono-infections and co-infections with *P*. *falciparum*. The low sensitivity of sensitivity of microscopy has been reported in western Kenya [2]. The routine microscopy severely underestimates the burden of infections as the submicroscopic infection may act as a reservoir of infectious gametocytes [56] and in some instances may develop to clinical infection [57–59]. The findings of these four-season cross-sectional surveys revealed that topography and seasonality contribute to persistent malaria transmission and have an impact on susceptibility to submicroscopic infection.

## Conclusion

A substantial proportion of submicroscopic malaria carriers were found in the population living along lakeshores and in mud houses, as well as among males and school-aged children. However, bed net use and population occupation had no effect on submicroscopic infection. To maintain progress toward parasite reservoir elimination and case malaria management, strategies for identifying malaria transmission determinants within sub-counties should be developed to guide the formulation of targeted interventions in different eco-epidemiological settings.

## Supporting information

S1 Table Multiple sub-microscopic infection comparisons within topographical zones. Dependent Variable: sub-miscroscopic. Tukey HSD. Based on observed means. The error term is Mean Square (Error) = .120.*. The mean difference is significant at the 0.05 level.
(DOCX)

S2 Table Predictive factors associated with submicroscopic infection in malaria infection. (DOCX)
