# Supplementary material for: Risk associations of submicroscopic malaria infection in lakeshore, plateau and highland areas of Kisumu County in western Kenya
Source: PLoS One. 2022 May 16;17(5):e0268463. doi: 10.1371/journal.pone.0268463 (PMC9109926; doi:10.1371/journal.pone.0268463)
Supplement: S1 Table — Dependent Variable: sub-miscroscopic. Tukey HSD. Based on observed means. The error term is Mean Square (Error) = .120.*. The mean difference is significant at the 0.05 level. (DOCX) [file pone.0268463.s001.docx]

**S1Table. Multiple submicroscopic infection comparisons within topographical zones**

| (I) Topography | (J) Topography | Mean Difference (I-J) | Std. Error | Sig. | 95% Confidence Interval | |
| --- | --- | --- | --- | --- | --- | --- |
|  |  |  |  |  | Lower Bound | Upper Bound |
| Lake | Slope | .07^*^ | .020 | .001 | .02 | .12 |
|  | Plateau | .13^*^ | .020 | .000 | .08 | .17 |
| Slope | Lake | -.07^*^ | .020 | .001 | -.12 | -.02 |
|  | Plateau | .06^*^ | .020 | .013 | .01 | .11 |
| Plateau | Lake | -.13^*^ | .020 | .000 | -.17 | -.08 |
|  | Slope | -.06^*^ | .020 | .013 | -.11 | -.01 |

Dependent Variable: submiscroscopic. Tukey HSD

Based on observed means.

The error term is Mean Square (Error) = .120.

*. The mean difference is significant at the .05 level.
