# Supplementary material for: Risk associations of submicroscopic malaria infection in lakeshore, plateau and highland areas of Kisumu County in western Kenya
Source: PLoS One. 2022 May 16;17(5):e0268463. doi: 10.1371/journal.pone.0268463 (PMC9109926; doi:10.1371/journal.pone.0268463)
Supplement: S2 Table — (DOCX) [file pone.0268463.s002.docx]

**S2 Table. Predictive factors associated with submicroscopic infection in malaria cases**

| Risk ratio | Category | Coefficient | AOR (95% CI) | Sig. |
| --- | --- | --- | --- | --- |
| Topography of residence area | Lakeshore | -0.247 | 0.78 (0.31, 1.95) | 0.596 |
|  | Hillside | -0.015 | 0.99 (0.36, 2.69) | 0.977 |
|  | Plateau |  | 1 |  |
| Age group | <5 years | -2.636 | 0.07 (0.03, 0.21) | <0.0001 |
|  | 5-<15 years | -1.314 | 0.27 (0.11, 0.67) | 0.005 |
|  | ≥15 years |  | 1 |  |
| Sex | Female | 0.574 | 1.78 (0.95, 3.33) | 0.073 |
|  | Male |  | 1 |  |
| Bed net usage | No net | 0.226 | 1.25 (0.33, 4.82) | 0.742 |
|  | Use net |  | 1 |  |
| Wall type | Brick & block | -0.3 | .74 (0.23, 2.36) | 0.611 |
|  | Mud & wood | -0.425 | 0.65 (0.28, 1.52) | 0.324 |
|  | Mud & cement |  | 1 |  |
| Occupation/ income generating activity | Farmer | -0.177 | 0.84 (0.29, 2.39) | 0.741 |
|  | Commercial sales | 0.887 | 2.43 (0.50, 11.75) | 0.270 |
|  | Child younger than working age | 0.667 | 1.95 (0.57, 6.71) | 0.290 |
|  | Unemployed |  | 1 |  |
| Symptoms | Asymptomatic | -0.187 | 0.83 (0.36, 1.90) | 0.659 |
|  | Fever |  | 1 |  |
| Seasonality | Wet (June 2019) | -0.475 | 0.62 (0.25, 1.57) | 0.316 |
|  | Dry (November 2019) | -0.331 | 0.72 (0.28, 1.85) | 0.493 |
|  | Wet (June 2020) | -0.966 | 0.38 (0.10, 1.40) | 0.145 |
|  | Dry (November 2020) |  | 1 |  |
